# The Natural History of Cervical Spondylotic Myelopathy and Ossification of the Posterior Longitudinal Ligament: A Review Article

**DOI:** 10.7759/cureus.5074

**Published:** 2019-07-03

**Authors:** Zaid Aljuboori, Maxwell Boakye

**Affiliations:** 1 Neurosurgery, University of Louisville School of Medicine, Louisville, USA

**Keywords:** spine, myelopathy, opll, compressive, spondylosis

## Abstract

Cervical spondylotic myelopathy (CSM) is the chronic and slow deterioration of cervical spinal cord function. The pathophysiology of this condition is multifactorial, including compression, repetitive trauma, and vascular compromise of the spinal cord. Clinically, it presents as a progressive decline in patients’ appendicular neurological function. The natural history of this disease varies but, it is well-known that the duration and degree of compression correlate negatively with prognosis. A mild degree of CSM tends to stabilize with potential improvement over time while more severe CSM tends to progress. Surgical intervention has shown to positively alter the natural history of the disease by halting the progression with some restoration of function.

Ossification of the posterior longitudinal ligament (OPLL) is a chronic disease that results in progressive ossification of the posterior longitudinal ligament of the spine. It commonly affects the cervical spine. The etiology is multifactorial in nature, including genetic and environmental factors. The progressive nature of this condition and the resultant cervical spinal stenosis make it one of the main causes of cervical myelopathy (CM). There is no medical therapy for this disease, and surgery is reserved for patients with CM caused by spinal cord compression. In this article, we review the different aspects of the natural history of both CSM and OPLL.

## Introduction and background

Cervical spondylotic myelopathy (CSM) is a clinical syndrome due to the disturbance of spinal cord function, both sensory and motor systems, due to a chronic degenerative process that results in progressive narrowing of the cervical spinal canal and resultant cord compression [1-3]. It usually presents as paresthesia, progressive loss of fine motor function, and imbalance. In advanced cases, patients do experience bowel and bladder dysfunction. The degenerative changes are intervertebral disc degeneration, osteophyte formation, and ligamentum flavum and facet hypertrophy [1-2,4]. The compression could be limited to a single level or may span multiple levels. It is the most common form of myelopathy in adults, especially in patients above 50 years of age [2,4]. The spinal canal narrowing predisposes the patient to chronic mechanical compression and vascular compromise of the spinal cord with additional repetitive trauma from the dynamic movement of the spine [3,5] These insults result in degeneration of the spinal cord [5-6].

Ossification of the posterior longitudinal ligament (OPLL) is a hyperostotic condition of the spine, where the posterior longitudinal ligament (PLL) becomes progressively calcified, often leading to symptomatic spinal canal stenosis [7-9]. Genetic, environmental, and biochemical factors have been implicated in the development of this disease with high prevalence in the Asian population [9-11]. OPLL also has familial distribution, it was radiographically evident in about a quarter of second-degree relatives, with a higher percentage in the siblings of OPLL patients [12]. It is considered a subtype of diffuse idiopathic skeletal hyperostosis (DISH) and can affect as many as half of the patients with DISH [10-11]. It is also prevalent among patients with cervical myelopathy [10]. There are four types of OPLL: 1) segmental, limited to the posterior surface of the vertebral bodies without crossing disc spaces; 2) continuous, spanning multiple levels with involvement of vertebral body and disc spaces; 3) mixed, a combination of segmental and continuous; and 4) other, limited to posterior disc spaces with some extension to the posterior vertebral body endplate [13-15].

## Review

Pathophysiology

Cervical Spondylotic Myelopathy (CSM)

CSM is a multifactorial process involving static (stenosis) and dynamic (repetitive trauma to the cord during spine movement) factors [1]. Congenitally short pedicles, bulging intervertebral discs, and osteophyte formation are examples of static factors causing stenosis of the spinal canal [1]. Movement of the cervical spine leads to dynamic changes in the spinal cord; during extension, the cord becomes thicker and shorter, which exaggerates the existing compression, while flexion leads to the narrowing and draping of the spinal cord over osteophyte ridges. These dynamic changes are thought to cause CSM through increasing tension on the spinal cord [16]. In addition, Breig et al. suggested that vascular compromise due to mechanical compression of the arterioles supplying the spinal cord contributes to the development of CSM [16]. Cadaveric studies showed that the degree of compression correlates with the pathologic changes involving the gray matter (neuronal loss and necrosis) and white matter (demyelination and gliosis) of the spinal cord [5-6,17]. Ogino et al. published an autopsy report on nine patients with a history of CSM with an average duration of symptoms of 18.2 yrs. They showed a positive correlation between the anteroposterior (AP) compression ratio of the spinal cord, with histopathological changes of the cord substance. At AP compression ratios of 40%-44%, 22%-39%, and 12%-19%, there was mild demyelination, gray matter cavitation with diffuse demyelination, extensive necrosis of gray matter, and gliosis of white matter, respectively [6].

These findings correlate with results from animal studies with experimental spinal stenosis and cord compression that demonstrated pathological changes mainly involving the gray matter with significant neuronal loss at the maximum area of compression [18].

Ossification of the Posterior Longitudinal Ligament

The pathophysiology of OPLL is poorly understood. Multiple studies have indicated an underlying genetic etiology [9-10,13,19-20]. The Investigation Committee on Ossification of the Spinal Ligaments in Japan performed a study on eight monozygotic and two dizygotic twins with OPLL. They reported that 75% of monozygotic twins had OPLL, which indicated the involvement of genetic factors [15]. Nakajima et al. conducted a genetic study comparing patients with OPLL and healthy controls (1130 vs 7135) using genome-wide association. The authors found multiple genes associated with OPLL. These genes are Radial Spoke Head 9 Homolog (RSPH9), Serine/Threonine Kinase 38 like (STK38L), Hydroxyacid Oxidase 1 (HAO1A), Coiled-Coil Domain Containing 91 (CCDC91), and R-Spondin 2 (RSPO2). The RSPH9 and STK38L genes might be responsible for the membranous ossification of the PLL. Additionally, the HAO1A, RSPO2, and CCDC91 genes are responsible for the endochondral ossification [21]. A genome-wide linkage study by Karasugi et al. of 214 Japanese affected sib-pairs showed that several loci on chromosomes 1p, 2p, 7q, 16q, and 20p were associated with OPLL. Of note, sib-pair is a linkage analysis of a dichotomous trait. It’s a common method used to narrow search results for genes that influence complex diseases. Additionally, microsatellite markers mapping showed a significant association with locus D20S894 on Ch. 20p12 [22]. In addition, epidemiological studies have shown that adult-onset obesity and type 2 diabetes mellitus (DM) are independent risk factors for OPLL [23-24]. Elevated serum insulin has been associated with increased ossification in patients with OPLL. The exact mechanism is unclear, but it is thought that insulin might stimulate osteoprogenitor cells, which results in the ossification of the PLL [24-25]. Sleep duration and dietary habits have been associated with n increased risk of OPLL as well [26-27]. Sleeping less than five or more than nine hours has been associated with increased risk of OPLL. High salt and low protein diet have been associated with OPLL as well but not cigarette smoking or alcohol consumption [26-27].

Natural history

Cervical Spondylotic Myelopathy

The natural history of CSM is described as a progressive worsening of signs and symptoms over time, but the rate and pattern of decline are unclear [1]. Two main patterns of CSM progression have been reported: (1) a slow worsening of function over time and (2) an extended period of stability of neurological function followed by expedited decline [28-29]. Clark et al., in their retrospective series of patients with CSM, observed that 75% of patients had a stable neurological function with episodic worsening while 20% had a slow progression over time [28]. Kadanka et al. evaluated the natural history of mild CSM modified Japanese Orthopedic Association score (mJOA) >12 treated either conservatively or surgically. The conservative group had 33 patients younger than 75 years with a one-year average duration of symptoms. At six months follow-up, 27% remained stable while 72% improved. At three years follow-up, 26% remained stable and 72% improved on the mJOA scale [30].

In addition to sensory and motor dysfunction, CSM has been shown to affect respiratory function. Bhagavatula et al., in their prospective study of pulmonary function test in 30 patients with CSM, showed that mean forced vital capacity (FVC) was 65%, which was significantly lower than in the healthy control group 88%. The mean forced expiratory volume in 1 second (FEV1) was 72% in the CSM group and 96% in the control group. The peak expiratory flow rate (PEFR) was 56.6 % in the CSM group and 68.3% in the control group. Additionally, there was a negative correlation between FVC and the Nurick score. [31] The exact mechanism for this subclinical respiratory dysfunction is not well-understood, but it was postulated that sensory, motor, and autonomic neural supply to the upper thorax through the C5-T1 nerve roots is the reason for this phenomenon [32].

Researchers have attempted to predict the development of CSM in patients with cervical stenosis using different strategies such as electrophysiological studies (EPS) and magnetic resonance spectroscopy (MRS). Kadanka et al., in their series of 30 patients with imaging-confirmed cervical spinal stenosis with no clinical evidence of CSM, performed a battery of electrophysiological studies including (motor-evoked potentials and sensory-evoked potentials). Fifty percent of patients had abnormal EPS; of this group, 30% percent developed CSM during the two years follow-up while patients with normal EPS did not develop CSM [33].

MRS has been used to detect early cellular biochemical changes in the spinal cord in patients with advanced cervical spondylosis. Salomon et al. conducted a comparison study of patients with cervical spondylosis and healthy controls using regular magnetic resonance imaging (MRI) and MRS. They found that the relative concentration of choline to creatine (Cho/Cr) was significantly lower in healthy controls than in cervical stenosis patients with T2 cord signal change. There was no significant difference in Cho/Cr between healthy controls and patients with cervical stenosis, with no T2 cord signal change. They also observed that the choline to N-Acetyl aspartate ratio (Cho/NAA) was significantly lower in the healthy controls in comparison to patients with cervical stenosis with or without T2 signal changes on MRI. They found a correlation between a lower Cho/NAA and higher mJOA score. They concluded that MRS can be used as a tool to detect early and late cellular biochemical changes of the spinal cord in patients with advanced cervical spondylosis. The choline/NAA ratio correlation with the mJOA score can provide a clinically useful radiographic biomarker to help manage patients with cervical spondylosis [34].

Roberts et al. conducted a retrospective study of 24 patients with a diagnosis of CSM based on clinical exam and myelography. They reported that the longevity and severity of symptoms correlated negatively with clinical improvement. They also reported that a symptom duration of more than 18 months was associated with a poor outcome [35]. Another study of 22 patients with a diagnosis of CSM by Sadasivan et al. showed that the longevity of symptoms prior to diagnosis affected outcomes. The patients in this study had symptoms for an average of 6.3 years before diagnosis; follow-up results showed that all patients deteriorated clinically [36].

Surgical decompression was proposed as a method to halt the progression of CSM, as well as to help patients by improving their neurological function and quality of life. Liu et al. conducted a study on 94 patients with CSM. They used a combination of the presence or absence of cord signal on MRI T2-weighted images along with n electromyography (EMG) analysis to predict functional outcome after surgical intervention. The EMG was either positive or negative. Positive results included lengthened duration (exceeding the normal value by 20% in the same age group), large-amplitude motor unit action potentials (MUAPs), and polyphasic motor units in at least two myotomes unilaterally or bilaterally. These electrophysiologic changes indicate anterior horn cell lesions. They stratified patients into four groups based on MRI and EMG results: (-/-), (+/-), (-/+), and (+/+). Patients with no MRI cord signal and negative EMG had the best outcomes while patients with MRI cord signal and positive EMG had the worst outcome [37]. In addition, Hirai et al. performed a comparative study of anterior cervical decompression with fusion (ADF) and posterior decompression using laminoplasty. Seventy-nine patients received either ADF or laminoplasty with two years follow-up. ADF resulted in a higher mean JOA recovery rate at two years in comparison to laminoplasty. One potential limitation of this study is the average duration of symptoms before surgery was longer for the laminoplasty group (22.8 vs 39.8 months) [38]. Chagas et al. also evaluated the benefit of anterior decompression and fusion (ADF) in CSM patients; 39 patients with a mean duration of symptoms before surgery of 38.1 months were treated with a minimum follow-up of 18 months. Results showed that 64.1% of the patients had an improved Nurick scale postoperatively, 33.3% remained the same, and 2.6% got worse. They also found that patients younger than 60 years of age were more likely to benefit from surgery as compared to patients of age more than 60 years [39].

Manzano et al. compared expansile cervical laminoplasty (ECL) and cervical laminectomy and fusion (CLF) for multilevel cervical myelopathy. They enrolled 16 patients with a mean age of 59 years (range 41-75 years); nine patients randomized to the ECL group and seven patients to the CLF group. The duration of symptoms was 17 months for the ECL group and 20 months for the CLF group. Both groups were evaluated using the mJOA and Nurick myelopathy scales at baseline and after surgery. Postoperatively, both groups had an improvement in both the mJOA and Nurick scales, but only the Nurick scale improvement was statistically significant. In the ECL group, the mJOA score improved from 12.37 +- 1.2 to 14.25 +- 0.96 while in the CLF group, the mJOA group improved from 12.57 +- 1.09 to 13.57 +- 1.02. They also reported that there were no significant differences in preoperative self-reported indexes between the two groups. Postoperatively, only the ECL group showed a statistically significant improvement in self-reported outcome measures, including neck, interscapular, and arm pain one year after surgery. The authors concluded that both procedures resulted in statistically significant improvements in neurological function. However, the ECL group had improvements in several self-reported outcome measures [40].

Fehlings et al. recently published clinical guidelines on the management of CSM. Their recommendations were as follow: (1) Surgical intervention is recommended for patients with moderate or severe CSM. (2) Patients with mild CSM can be offered surgical intervention or a trial of rehabilitation. Operative intervention should be considered if there is neurological deterioration or failure to improve with rehabilitation. (3) Prophylactic surgery should not be considered for patients with evidence of cervical cord compression but without myelopathy or signs and symptoms of radiculopathy. These patients should be counseled on the potential risks of progression and followed clinically. (4) Patients with evidence of cord compression and no myelopathy but have clinical evidence of radiculopathy are at a higher risk of developing myelopathy. These patients can be offered surgical intervention or a trial of rehabilitation with close follow-up. If the non-surgical option was pursued and the patients developed myelopathy, the patient should be managed according to the first or second recommendation depending on the severity of the clinical picture [41].

Ossification of Posterior Longitudinal Ligament

The natural history of OPLL has been well-studied, especially in the Asian population, researchers have evaluated radiographic progression, development of myelopathy, and the effects of different surgical techniques on the progression and clinical outcomes [13-14,42-43]. Katsumi et al. evaluated 41 OPLL patients with a mean age of 61.8 yrs, who were treated conservatively. The mean follow-up period was 25.6 +- 17.1 months. Each patient was evaluated with a computed tomography (CT) scan twice, with a one-year interval to assess the progression of the disease. Computer software was used to analyze the patients' CT images and create a 3D model of the OPLL. The study demonstrated that there was no effect of gender, DM, family history of OPLL, and OPLL type and location on the rate of lesion progression. Additionally, over the course of the follow-up period, the ossification volume increased by 7.5%. Interestingly, they described that the annual rate of progression of OPLL differs by age group, with the highest rate afflicting the 30-49 years' age group [14].

Matsunaga et al. evaluated the radiographic progression of OPLL on both the axial and longitudinal planes over time. They enrolled 167 patients with OPLL, who were treated conservatively with a mean follow-up of 11.2 years. Forty-two percent of patients had axial progression; of these, 45% had more than a 2 mm increase in thickness while the rest had a less than 2 mm increase. Eighty-two percent of patients had an extension of the OPLL along the longitudinal plane [42]. In another study by Matsunaga et al. of the effects of strain distribution in the intervertebral disc space on the progression of OPLL, they enrolled 101 patients with OPLL of the cervical spine with no previous surgery. Dynamic lateral X-rays and a computer software program were used to analyze the strain distribution. They categorized the patients according to the type of OPLL: 23 continuous type, 25 mixed type, 49 segmental type, and 4 other types. Fifty-two (51%) patients had progression over time. They found abnormal strain distribution in 62% (63) of the patients. Seventy-one percent (45) of this group showed the progression of ossification that matched the areas of abnormal strain distribution while only 18% of patients without abnormal strain distribution showed progression of OPLL [44].

Several studies have evaluated the development of cervical myelopathy in patients of OPLL. A multicenter cohort study was performed by the investigative committee on OPLL of the Japanese Ministry of Public Health, Labor, and Welfare to study the radiographic predictors for the development of myelopathy in patients with OPLL. They used X-rays, CT, and MRI. A total of 156 patients were enrolled with a mean follow-up of 10.3 years. The authors classified the ossification on the axial plane into the central and laterally deviated types. Thirty-nine patients had more than 60% compromise of the spinal canal by OPLL; all of these patients had myelopathy while only 49% of the patients with less than 60% canal compromise by OPLL had myelopathy. Additionally, the authors evaluated the dynamic movement of the cervical spine and found that it was higher in patients with myelopathy. They concluded that a lateral deviated axial ossification pattern and canal compromise of more than 60% are radiographic predictors for myelopathy [45].

Additionally, Matsunaga et al. studied the development of myelopathy in 359 patients with OPLL, with a minimum of 10 years follow-up (average 17.6 years). Ninety percent of patients had no myelopathy at the time of diagnosis. Upon follow up, 17% developed myelopathy. Their analyses revealed that the myelopathy free rate was 71% at the 30 years follow-up in patients with OPLL and no myelopathy. Of the remaining 10% of the population with OPLL and myelopathy at the time of diagnosis, the majority had a worsening of their myelopathy without treatment. In this cohort, all patients with canal compromise of more than 60% had myelopathy. They also found that range of motion (ROM) was higher in patients with myelopathy and less than 60% canal compromise. They concluded that prophylactic surgery in patients with OPLL without myelopathy may not be necessary. In addition, dynamic factors are of importance in the development of myelopathy, especially in patients with less than 60% canal compromise [43].

In regard to the effects of surgical intervention on the natural history of OPLL, Ogawa et al. retrospectively reviewed 72 patients with OPLL myelopathy who underwent open door laminoplasty with a minimum follow-up of five years. The mean preoperative JOA score was 9.2 +- 0.4. At the three years follow-up, the score improved to a mean of 14.2 +- 0.3. This improvement was maintained at the five-year time point. They also reported postoperative progression of more than 2 mm of OPLL in 63.9% of the patients. In the axial plane OPLL thickness increased by a mean of 3.9 mm, and in the longitudinal plane by a mean of 26.3mm. They reported late onset deterioration in 11 patients, which occurred more than 10 years after surgery [46]. Kawaguchi et al. described an association between laminoplasty done for OPLL myelopathy and the radiographic progression of OPLL. In their study, 73% of patients had radiographic progression of OPLL after surgery. The longitudinal progression was worse than the axial progression. They also noticed that patients who had progression were younger (mean 53.3 years) than the non-progression group (mean 60.2 years). In addition, there was an association between the mixed or continuous type of ossification and the radiographic progression of OPLL [47]. Li et al. systematically reviewed studies that involved patients undergoing surgical decompression for OPLL myelopathy. They reported surgical complications, which included spinal cord injury, cerebrospinal fluid (CSF) leak, implant failure. CSF leak, hoarseness of voice, dyspnea, and dysphagia were more common with anterior approaches [48].

CT imaging has a valuable role in predicting the risk of a CSF leak in anterior approaches. It can be used to detect dural defects. The double-layer sign on CT describes the anterior and posterior rims of hyperdense regions (calcification) separated by a hypodense area (dural defect) due to OPLL growth into the dura [49].

Since the increased mobility caused by posterior surgical approaches resulted in a higher progression rate of OPLL in comparison to anterior approaches, an effort was made to minimize the complications associated with anterior approaches. The anterior floating method was described where a thin layer of OPLL was left adherent to the dura during corpectomy to avoid complications like a CSF leak. Matsuoka et al. studied the effects of anterior floating method decompression and fusion on 63 patients with cervical OPLL with a follow-up of more than 10 years. They reported that only three patients had a progression of OPLL (1-1.5 mm) at the operative site. Two of these three were attributed to inadequate thinning of the OPLL, and the third one was attributed to the side residual of OPLL. In 36 patients, there was a progression of OPLL in the segments adjacent to the operative site [50].

## Conclusions

CSM is a chronic disease with different patterns of progression. It affects both the gray and white matter of the spinal cord, which leads to dysfunction of both motor and sensory systems. In addition, it affects the respiratory function of the patients. The length and severity of symptoms correlate negatively with prognosis. Surgical decompression may help halt disease progression and lead to variable improvement of function. OPLL is a multifactorial disease characterized by the chronic and progressive ossification of the PLL. Multiple genetic and biochemical aberrations have been linked to the development of this condition. Over time, it often leads to compressive myelopathy, which may require surgical intervention. Both the anterior and posterior surgical decompression approaches have been described, but the posterior approaches are more favored due to a lower complication rate.
